# Technical Note: Comprehensive performance tests of the first clinical real‐time motion tracking and compensation system using MLC and jaws

**DOI:** 10.1002/mp.14171

**Published:** 2020-05-11

**Authors:** Guang‐Pei Chen, An Tai, Timothy D. Keiper, Sara Lim, X. Allen Li

**Affiliations:** ^1^ Department of Radiation Oncology Medical College of Wisconsin 8701 Watertown Plan Rd Milwaukee WI 53226 USA

**Keywords:** motion tracking, synchrony, tomotherapy

## Abstract

**Purpose:**

To evaluate the performance of the first clinical real‐time motion tracking and compensation system using multileaf collimator (MLC) and jaws during helical tomotherapy delivery.

**Methods:**

Appropriate mechanical and dosimetry tests were performed on the first clinical real‐time motion tracking system (Synchrony on Radixact, Accuray Inc) recently installed in our institution. kV radiography dose was measured by CTDIw using a pencil chamber. Changes of beam characteristics with jaw offset and MLC leaf shift were evaluated. Various dosimeters and phantoms including A1SL ion chamber (Standard Imaging), Gafchromic EBT3 films (Ashland), TomoPhantom (Med Cal), ArcCheck (Sun Nuclear), Delta4 (ScandiDos), with fiducial or high contrast inserts, placed on two dynamical motion platforms (CIRS dynamic motion‐CIRS, Hexamotion‐ScandiDos), were used to assess the dosimetric accuracy of the available Synchrony modalities: fiducial tracking with nonrespiratory motion (FNR), fiducial tracking with respiratory modeling (FR), and fiducial free (e.g., lung tumor tracking) with respiratory modeling (FFR). Motion detection accuracy of a tracking target, defined as the difference between the predicted and instructed target positions, was evaluated with the root mean square (RMS). The dose accuracy of motion compensation was evaluated by verifying the dose output constancy and by comparing measured and planned (predicted) three‐dimensional (3D) dose distributions based on gamma analysis.

**Results:**

The measured CTDIw for a single radiograph with a 120 kVp and 1.6 mAs protocol was 0.084 mGy, implying a low imaging dose of 8.4 mGy for a typical Synchrony motion tracking fraction with 100 radiographs. The dosimetric effect of the jaw swing or MLC leaf shift was minimal on depth dose (<0.5%) and was <2% on both beam profile width and output for typical motions. The motion detection accuracies, that is, RMS, were 0.84, 1.13, and 0.48 mm for FNR, FR, and FFR, respectively, well within the 1.5 mm recommended tolerance. Dose constancy with Synchrony was found to be within 2%. The gamma passing rates of 3D dose measurements for a variety of Synchrony plans were well within the acceptable level.

**Conclusions:**

The motion tracking and compensation using kV radiography, MLC shifting, and jaw swing during helical tomotherapy delivery was tested to be mechanically and dosimetrically accurate for clinical use.

## 1 INTRODUCTION

Organ motion during radiation therapy (RT) delivery, caused primarily by either respiration and/or peristalsis, can be managed with a variety of techniques. These techniques include (a) planning target volume expansion,^1^ (b) motion reduction, such as breath holding^2^ and abdominal compression,^3^ (c) beam gating,^4^, ^5^ and (d) real time motion compensation.^6^ Real time motion compensation, arguably the most effective method, contains two distinct components: tumor motion monitoring and dose compensation. Motion monitoring can be implemented with external surrogates, implanted internal fiducial markers, or internal anatomic structures.^6^ A variety of methods of dose correction have been investigated, including radiation source tracking,^7^, ^8^ couch tracking,^9^, ^10^ robotic tracking, ^11^, ^12^ gimbal‐based dynamic tracking,^13^, ^14^, ^15^ and dynamic multileaf collimator (MLC) tracking.^16^ Multileaf collimator tracking is the most widely investigated method, where a treatment beam is continuously reshaped according to the target motion in beam’s eye view using MLC leaves. Multileaf collimator tracking was first suggested in 2001 and implemented with preprogrammed one‐dimensional motion compensation and manual synchronization,^17^ and has been demonstrated with real‐time feedback compensation on major LINAC platforms such as Varian,^18^, ^19^, ^20^, ^21^ Siemens,^22^, ^23^ and Elekta.^24^, ^25^

Recently, a real time motion monitoring and compensating system, Synchrony on Radixact (Accuray Inc, Sunnyvale, CA),^26^ was introduced based on the successful robotic tracking system, Synchrony on CyberKnife (Accuray Inc).^27^ This Synchrony on Radixact system utilizes rotational two‐dimensional (2D) kV x‐ray radiograph for real‐time motion monitoring to instruct binary MLC and jaws for motion compensation during helical tomotherapy delivery.^28^, ^29^ The system is, so far, the only commercially available system with combined jaw and MLC tracking.

The first clinical Synchrony on Radixact system was installed recently at Froedtert & Medical College of Wisconsin.^30^ Prior to the clinical use of the system, commissioning and quality assurance (QA) tests were performed in order to verify the mechanical and dosimetric performance of the system. These tests investigate the effect of jaw and MLC motions on the characteristics of the treatment beams and the efficacy of jaw and MLC tracking which is subject to the spatial and temporal resolution of the target localization and system lag times. Details of these tests and key results are presented.

## 2 MATERIALS AND METHODS

### 2.A. Methods of motion tracking and compensation

Our existing Radixact machine (Accuray Inc.) was upgraded to include a kV x‐ray imaging system mounted 90˚ offset from the MV treatment beam, an optical camera system mounted on the ceiling above the foot of the couch, and a new control software package. By utilizing these additional components, target motion can be detected and predicted to instruct jaw swing and MLC leaf shift to synchronize with the target motion. The system supports three synchronization modes based on the object to be detected on the radiograph images as well as the type of tumor motion:

- Fiducial tracking for nonrespiratory (irregular) motion (FNR), for example, prostate motion,
- Fiducial tracking for respiratory motion (FR),
- Fiducial free tracking (e.g., lung tumor) for respiratory motion (FFR).

In the planning stage, digitally reconstructed radiographs (DRR) are generated from the planning image at 2 to 6 specified acquisition angles depending on the visibility of the tracking target and mechanical limitations. After patient (phantom) setup, radiographs are collected at the prescribed angles with a gantry period of 10 s and a static couch positioned at the initial treatment position. Target (tumor or fiducials) motion is measured and monitored by comparison of the tracking targets on the kV radiographs and the DRRs to determine the motion from the baseline. During treatment, the radiograph images are acquired according to the planned gantry rotation and couch movement.

For respiratory (periodic) motion, four LED markers are used as external surrogates; three are attached to the patient and one is fixed on the couch. An internal–external correlation model^28^ is built between the internal target position, visible in the radiograph images, and LED marker positions, detected continuously by the camera. This correlation model is updated with newly acquired radiograph images and used to continuously (every 10 ms) predict the target position in 3D. For nonrespiratory motion, no external surrogate is used, instead, a statistical position model^28^ is generated with acquired sequential 2D radiograph images to model discrete translation of the 3D target positions. The model‐predicted target position at the current radiograph image is used until the next radiograph image is acquired. For either respiratory or nonrespiratory motion, the motion compensation is implemented with jaw swing for longitudinal motion (IEC Y) and MLC leaf shifting for lateral (IEC X) and vertical (IEC Z) motions following the predicted tumor motion. The tumor is assumed to be rigid, that is, there is only beam repointing (no reshaping) for dose compensation. Because the jaws require room to swing, tracking is available for two of three available jaw widths, 1.0 and 2.5 cm.

A tracking range around planned fiducial or tumor locations on DRRs is used to limit the searching window to detect the fiducial or tumor position on the radiograph image. If the search is successful, several parameters are calculated to evaluate the goodness of the radiograph image against their preset thresholds. These parameters include the rigidity of the target with fiducials implanted (Rigid Body), the maximum predicted variance in the 3D model positions for the next model build (Potential Diff), the 2D distance between the predicted and detected fiducial or target positions (Measured Δ) and the 3D distance between the detected and planned fiducial or target positions (Target Offset). Safety considerations warrant a few scenarios which will cause automatic interruption of the delivery. Irradiation will pause if the duration between high‐confidence detection on sequential radiographs reaches a preset value. Treatment can be resumed after additional radiographs are acquired and the model parameters are met. Due to the limitation of jaw swing range, treatment will stop if the tumor moves out of the reach of jaw swing for a certain amount of time specified by the user. If the center of tumor motion has a systematic offset in IEC Y, the center of motion deviation is calculated to determine if it would be helpful to move couch in Y direction so that the jaw swing can cover the tumor motion range. The motion monitoring during the treatment delivery includes graphical presentations of the LED amplitudes, comet graphs representing model points derived from radiographs overlapped with the model prediction, the predicted offset in three directions (IEC X, Y, Z) as well as their vector magnitude.

### 2.B. Imaging dose and image quality for motion monitoring

**Fig. 1:**
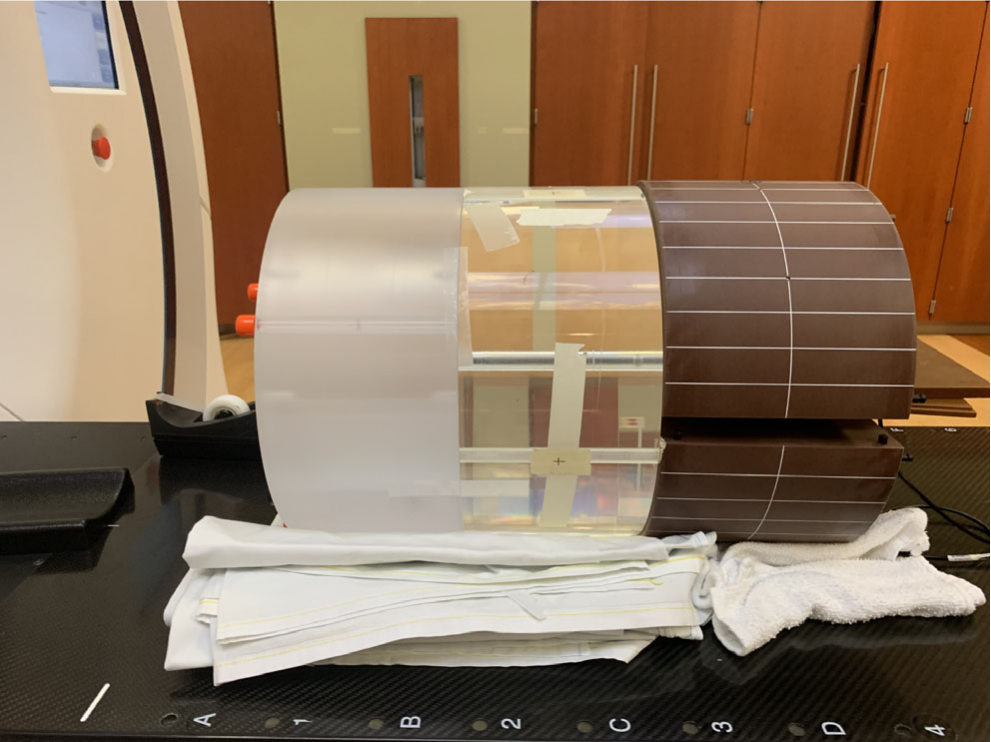
Phantom setup for CTDI measurements. [Color figure can be viewed at wileyonlinelibrary.com]

To account for imaging dose from the motion monitoring using kV radiography, we measured CTDI_100_ at the center and four peripheral positions^31^ using a Fluke 76‐415 CT body dose phantom of 32 cm in diameter and 15 cm in width and a pencil chamber (Fig. 1). The phantom was sandwiched by another CTDI phantom and the TomoPhantom to ensure sufficient scatter. The measurements were performed with the pencil chamber at center as well as at four peripheral positions (0°, 90°, 180°, and 270°) around the phantom for only kV radiography, that is, no MV beam delivery. A radiography protocol with 120 kVp and 1.6 mAs was used. Images were acquired for three rotations with six roughly evenly distributed image acquisition angles per gantry rotation in order to achieve a stable reading and two to three readings were obtained at each chamber position. Weight CTDI, CTDI_w_, was then calculated based on these measurements.

### 2.C. Effect of motion compensation on beam characteristics

**Fig. 2:**
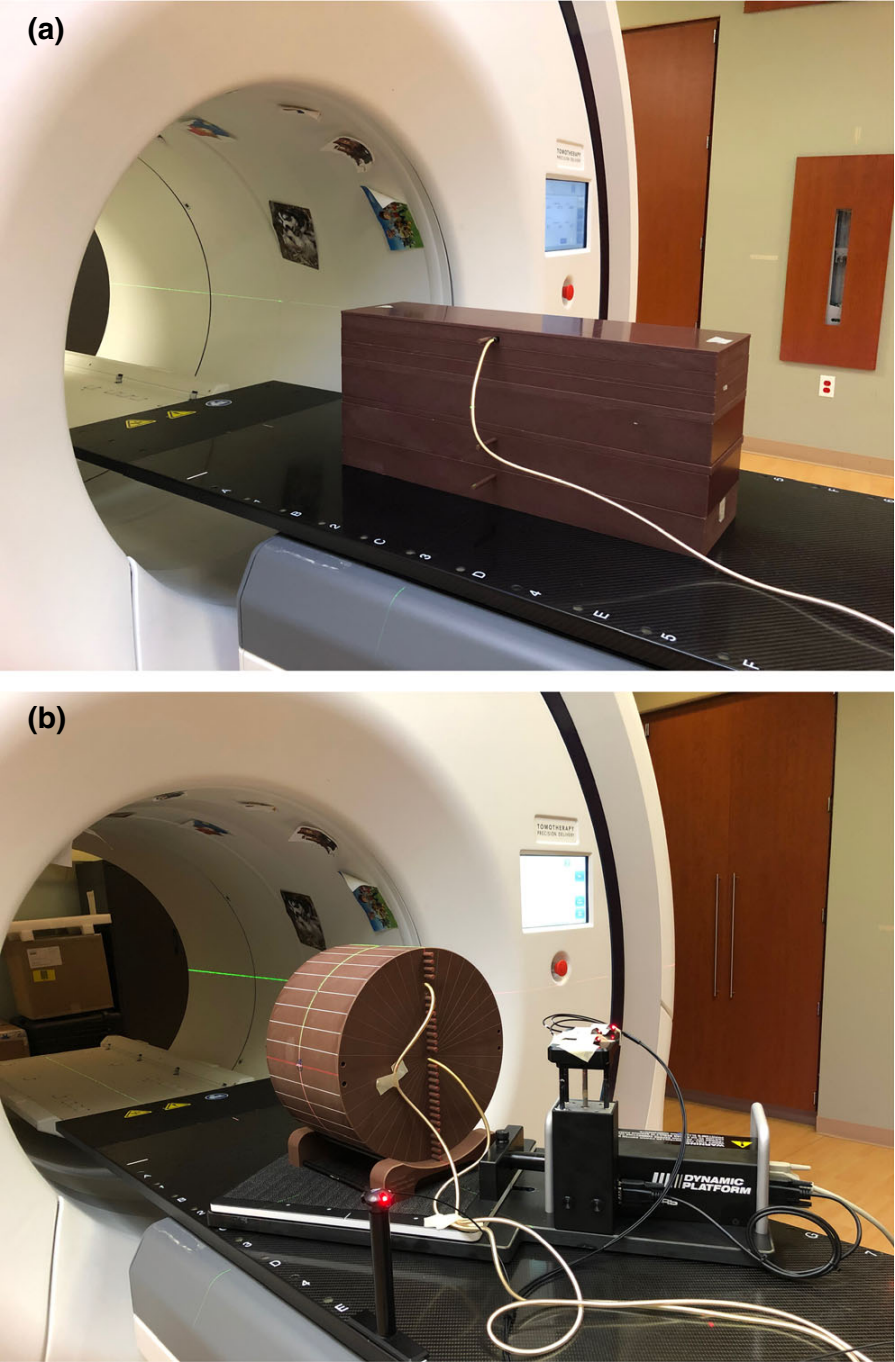
(a) Setup for longitudinal profile scans with blocks of rectangular virtual water. (b) Setup for ion chamber measurement. [Color figure can be viewed at wileyonlinelibrary.com]

As the motion tracking may use off‐axis beams due to the swinging of jaws and shift of MLC leaves, the effect of motion tracking on beam quality was measured. Rectangular virtual water slabs were positioned on the couch with the longest dimension oriented along IEC Y. An A1SL ion chamber inserted into virtual water at various depths was used to measure the longitudinal beam profile of a static beam at gantry angle 0˚. The virtual water slabs were setup with 85 cm source to surface distance [Fig. 2(a)]. The measurements were carried out for the 1.0 and 2.5 cm jaw openings with jaws moved to the negative extreme, center, and positive extreme positions. The extreme positions were ±2.0 cm from isocenter for the 1.0 cm jaws, and ±1.25 cm from isocenter for the 2.5 cm jaw. The beam characteristics at these extreme jaw positions were measured with open MLC. The measurements were performed for six jaw configurations at the following ion chamber locations: (a) the ion chamber was centered in IEC X and located at the depths of 1.0, 1.5, 5.0, 10.0, 15.0, and 20.0 cm; and (b) the ion chamber together with the virtual water slabs were offset in IEC X by −2.0 cm and located at the depths of 1.5, 15.0, and 20.0 cm. No measurements with the ion chamber offset to the positive IEC X direction were performed due to symmetry of the beam profile.

The peak value, $D(J,d,X_{\text{IC}},Y_{\text{Jaw}})$ , and the full width half maximum (FWHM), $W({J,d,X}_{\text{IC}},Y_{\text{Jaw}})$ , for each measured beam profile were collected. Here J is the jaw width, d is the ion chamber depth, $X_{\text{IC}}$ is the IEC X of the ion chamber, and $Y_{\text{Jaw}}$ is the IEC Y of jaw center. A jaw offset peak factor (JOPF) is defined as the ratio of the peak values when the jaw is shifted by $Y_{\text{Jaw}}$ to no swing, $Y_{\text{Jaw}}=0$ , both with the ion chamber centered in IEC X:

$${\text{JOPF}}_{J,Y_{\text{Jaw}}}\left(d\right)=\frac{D\left(J,d,0,Y_{\text{Jaw}}\right)}{D\left(J,d,0,0\right)}.$$

A jaw offset width factor (JOWF) is defined as the ratio of FWHM values for jaw swing at $Y_{\text{Jaw}}$ to no swing, $Y_{\text{Jaw}}=0$ , both with ion chamber centered in IEC X:

$${\text{JOWF}}_{J,Y_{\text{Jaw}}}\left(d\right)=\frac{W\left(J,d,0,Y_{\text{Jaw}}\right)}{W\left(J,d,0,0\right)}.$$

Accordingly, an MLC leaf shift peak factor (LSPF) is defined as the ratio of peak values with the ion chamber offset in IEC X by $X_{\text{IC}}$ to that without offset, $X_{\text{IC}}=X_{0}$ ,

$${\text{LSPF}}_{\text{MLC}}\left(X_{\text{IC}},X_{0},d\right)=\frac{D\left(J,d,X_{\text{IC}},0\right)}{D\left(J,d,X_{0},0\right)}.$$

For each jaw width, the variation of the JOPF, JOWF, and LSPF with depth were used to measure the effect of jaw swing on beam quality.

### 2.D. Dose measurement under motion compensation

Motion compensation plans (Synchrony plans) were created based on CT datasets of a cylindrical phantom (TomoPhantom, Accuray) with the ion chamber pegs oriented vertically on the negative IEC Y side and aligned to the room lasers [Fig. 2(b)]. The phantom was placed on a motion platform (CIRS, Model 008PL, Norfolk, VA) which was rotated 30˚ around IEC Z to allow motion prescription in 2D (IEC X and Y). Two tracking targets were considered, a gold fiducial marker implanted at the tip of an ion chamber plug for both FNR and FR tracking and a 1 cm diameter titanium rod embedded in a density plug as the tracking target for FFR tracking. Synchrony plans for each tracking method and each jaw width, together with corresponding non‐Synchrony plans, were generated. The geometry and dosimetry for each Synchrony and non‐Synchrony plan pair are identical. A vendor provided irregular motion pattern, with repeating sudden movement of up to ±10 mm followed by a gradual drift toward baseline, was used for FNR plan delivery, while Sine waves with frequency of 4 s and various amplitudes were used for FR and FFR plan delivery. No motion trace was used for the delivery of a non‐Synchrony plan, although the measurement setup was identical to the delivery of a Synchrony plan. To exclude the imaging dose of the kV radiography from the dose delivery, ion chamber measurements were performed for all Synchrony plans with MLC and jaws closed in the positive extreme position. The measured image dose was subtracted from the measurement of the Synchrony plan. The ratios of ion chamber measurements at the same location from both Synchrony and the corresponding non‐Synchrony plan deliveries were calculated to evaluate the dose output effect from the motion compensation.

### 2.E. Detection accuracy of tracking target

A Daily QA3 device (Sun Nuclear Inc), situated statically on the couch with a manual offset of −5 mm in both IEC X and Y compared to the planned FNR plan setup position, as confirmed by image‐guided radiotherapy (IGRT) registration, was used to verify the system’s capability to detect a static target.

To measure the target motion detection accuracy during motion tracking, Synchrony phantom plans of the three tracking methods were delivered to the TomoPhantom placed on the CIRS motion platform [Fig. 2(b)]. A variety of irregular and Sine motion traces with motion amplitude up to ±10 mm was used to drive the motion platform. After delivery, the predicted tracking target positions were read out from the raw data stored in the system and were compared with the input traces. The tracking accuracy was measured by the root mean square (RMS) of the difference between the tracked (predicted) and the driven motion of the phantom (instructed) positions with the number of data points taken from after interpolation to the desired temporal resolution (e.g., a resampling frequency of 30 Hz was used for all Synchrony plans in this study).

### 2.F. Residual latency

A correction mechanism based on linear extrapolation was built into the system to account for the motion correction latency (30 and 70 ms for jaws and MLC).^28^ As primary motion is commonly in the longitudinal (IEC Y) direction, we investigated the remaining, residual jaw correction latency. We generated an FFR plan for a cylindrical target inside the TomoPhantom with both long axes along IEC Y and through isocenter. We then delivered the plan with respiratory motion correction by using a sine wave motion trace with a period of 3 s and amplitude of ±10 mm. The measured front and back jaw positions, the so‐called J values (in nominal units), were used to calculate the jaw center positions, which were fit with a sine function:

$$P=a\cdot \sin\left[\frac{2\pi }{T}\cdot \left(t+t_{0}\right)\right]+b,$$

where *P* is the position at time *t*, *T* is the motion period, *t*_0_ is related to the initial phase, and *b* is the motion position offset. The predicted target positions in Y from the system, with internally synchronized timestamp, were also sampled and fit with the same sine wave. The difference between the *t*_0_’s from the two is then the residual jaw correction latency.

### 2.G. Dose accuracy of motion compensation

The center ion chamber in the Daily QA3 device was used to measure the daily machine output and its constancy. Gafchromic EBT3 films (Ashland, Covington, KY) from the same batch, sandwiched between the two halves of the TomoPhantom, were used to measure 2D dose distributions of a variety of Synchrony plans with either sine waves (motion amplitude of ±10 mm and frequency of 2.5 or 4 s) and realistic respiration motion patterns for tracking with respiratory modelling or an irregular motion trace (maximum motion of ±10 mm in one direction for the first half and then the other direction for the second half) for FNR. For comparison, corresponding non‐Synchrony plans with and without motion were also delivered and measured. Each of the films irradiated with non‐Synchrony plans was also irradiated with the kV radiographs from the corresponding Synchrony plan (without MV beam). All irradiated films were scanned, and the obtained isodose lines and profiles, for Synchrony vs non‐Synchrony without motion, and Synchrony vs non‐Synchrony with motion, were compared.

Two 3D dosimeters, a cylindrical diode array (ArcCheck, Sun Nuclear) and a pair of orthogonal diode arrays (Delta4 Phantom+, ScandiDos, Uppsala, Sweden) were used to measure 3D doses of both Synchrony and non‐Synchrony deliveries. The ArcCheck was placed on the CIRS motion platform rotated 30˚ and an antislip nylon piece was used to keep the dosimeter stable during platform motion. Due to the similarity of the diodes and fiducials in the radiographs, the ArcCheck was not used to measure fiducial tracking plans. Since diodes have a nonlinear response in the kV range,^32^ the contribution from the kV radiography, measured with radiographs taken at same couch positions and gantry angles but with MLC leaves and jaws closed, was subtracted. Gamma analysis using the TG218 recommended criteria^33^ of 2 mm, 3% with a 10% threshold was performed to confirm the dose delivery accuracy against the planned dose.

**Fig. 3:**
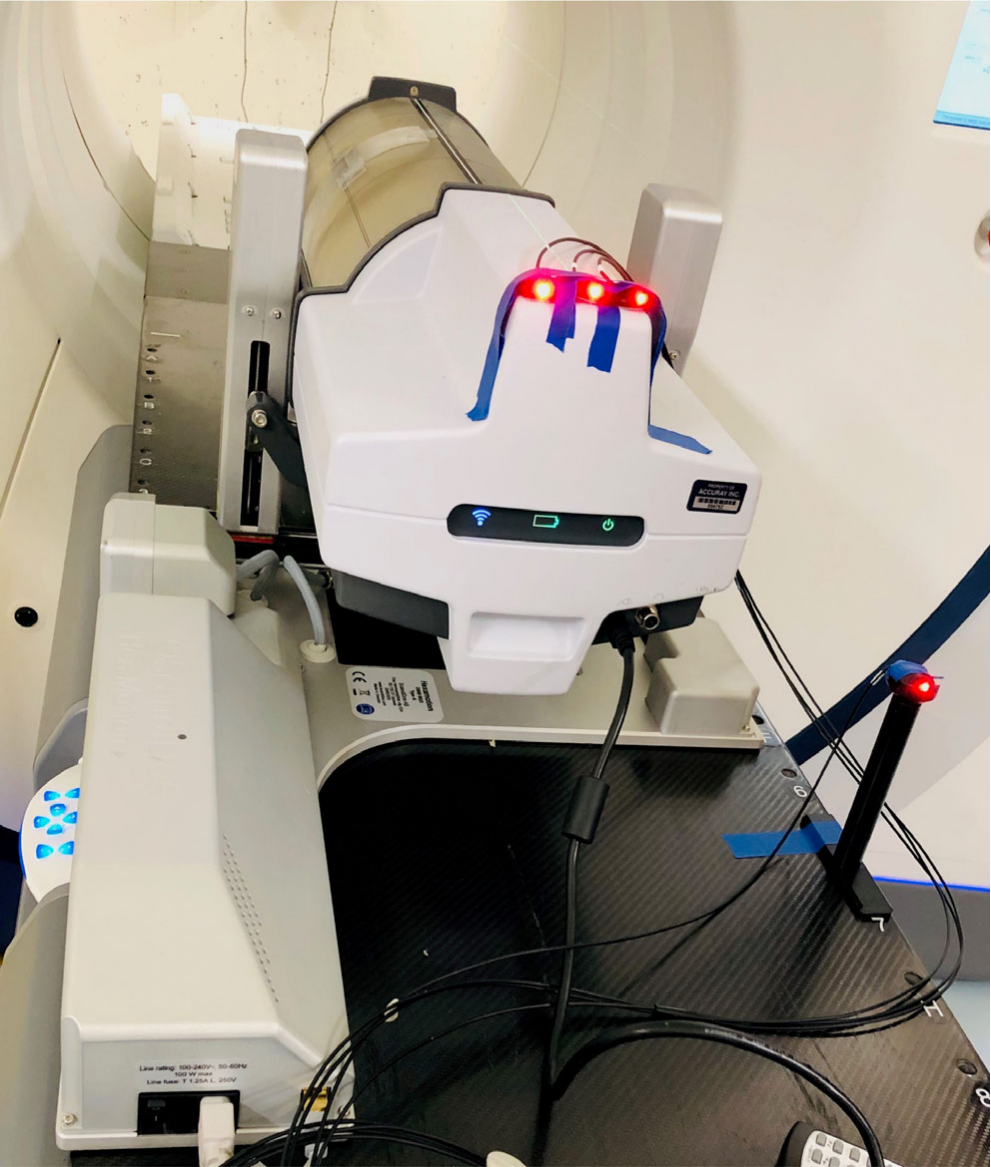
Delivery setup with Delta4 Phantom + on a HexaMotion six‐dimensional motion management system. [Color figure can be viewed at wileyonlinelibrary.com]

The Delta4 device placed on a 6D motion platform (Hexamotion, ScandiDos, Uppsala, Sweden) contained either a fiducial cube insert (for FNR and FR) or a lung cube insert (for FFR). The fiducial cube had six identifiable fiducials implanted, and the lung cube was comprised of a high‐density sphere embedded in a low‐density medium. Synchrony plans of FNR, FR, and FFR along with corresponding non‐Synchrony plans were delivered to the Delta4 phantom (Fig. 3). Contrary to those used for the CIRS dynamic phantom, motion traces used for the Hexamotion platform have independent IEC X/Y/Z motion. Similar to the ArcCheck measurements, radiograph doses were subtracted using the Delta4 software (ScandiDos Delta4) and the obtained delivery dose was compared with the plan dose. The same gamma criteria used in ArcCheck analysis were used.

In order to test the effectiveness of the “Move Couch in Y” feature, as well as the dose consistency when a delivery is interrupted and resumed, motion traces with baseline shifts were used for Synchrony plan delivery on the Delta4 device. Two plan deliveries each were carried out for FR and FNR, the first delivery for each tracking mode without interruption and the second delivery with manual corrections when the system detected a baseline shift consistent with the planned shift.

## 3 RESULTS

### 3.A. Imaging dose of kV radiography

The CTDI_w_ per projection was measured to be 0.084 mGy for the 120 kVp 1.6 mAs protocol. The value is less than the vendor quoted value of 0.16 mGy,^34^ which was measured in air. For a typical Synchrony delivery, approximately 100 radiographic imaging projections would be acquired, which corresponds to 8.4 mGy. This dose is substantially smaller than the imaging dose for regular IGRT (e.g., cone‐beam CT).

### 3.B. Effect of motion correction on beam characteristics

**Fig. 4:**
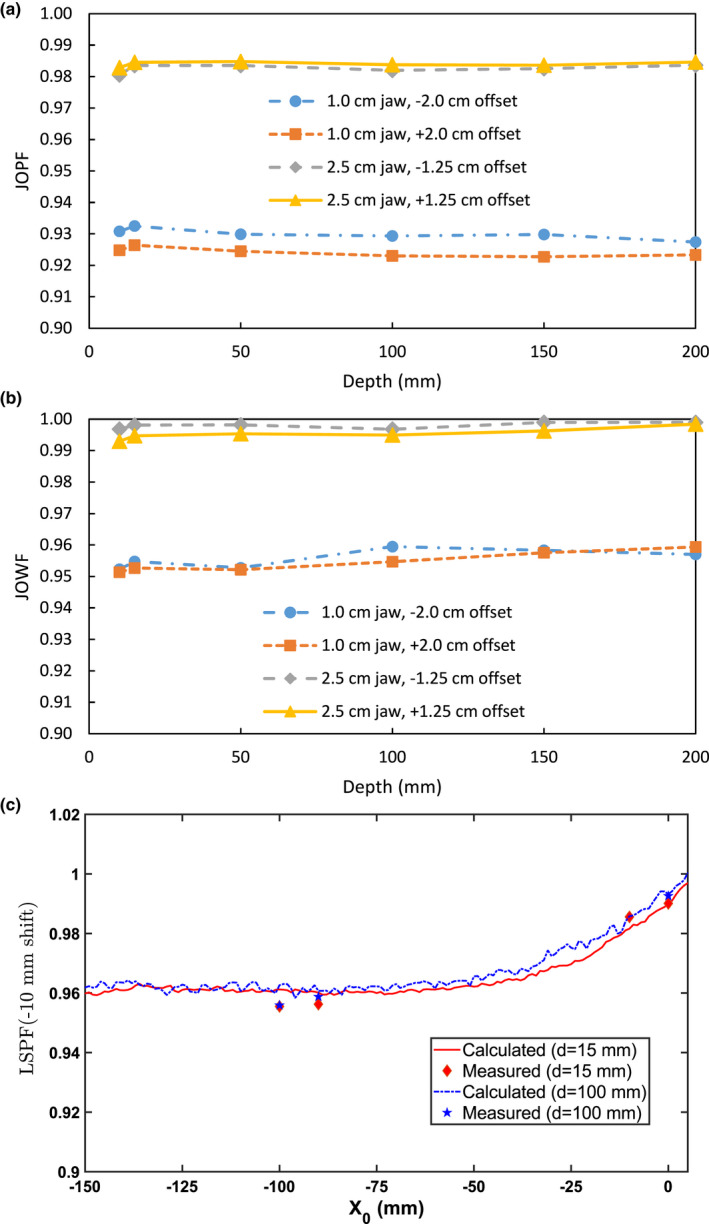
Variation of jaw offset peak factors (JOPFs) (a) and jaw offset width factors (JOWFs) (b) with depths. JOPF/JOWF are the ratios of the profile peak/width value when the jaw is shifted to that when the jaw is centered. (c) Calculated and measured LSPF values at 15 and 100 mm depths for 1 cm jaw with −10 mm lateral offset. [Color figure can be viewed at wileyonlinelibrary.com]

Figure 4 compares the characteristics of IEC Y off‐centered beams with depth. The horizontal trend seen for all curves in Fig. 4 demonstrate that the depth dependence of both JOPF and JOWF is small, indicating that the beam percentage depth doses (PDD) with an offset jaw are approximately the same as those of the centered jaw. However, delivery in extreme jaw positions results in a reduced dose to the target for both the 1.0 and 2.5 cm jaw widths. The average values, ${\bar{\text{JOPF}}}_{\text{1.0 cm, -2.0 cm}}=0.930\pm 0.002$ , ${\bar{\text{JOPF}}}_{\text{1.0 cm, +2.0 cm}}=0.924\pm 0.001$ , ${\bar{\text{JOPF}}}_{\text{2.5 cm, -1.25 cm}}=0.983\pm 0.001$ , and ${\bar{\text{JOPF}}}_{\text{2.5 cm, +1.25 cm}}=0.984\pm 0.001$ , show a more drastic dependence of the output on the IEC Y offset for the 1.0 cm jaws compared to the 2.5 cm jaws. As shown in Fig. 4(b), JOWFs vary slightly with depth for both 1.0 and 2.5 cm jaws. The average JOWF for the 1.0 cm jaw was 0.955 with a standard deviation (SD) of 0.003, and the average JOWF for the 2.5 cm jaw was 0.997 with an SD of 0.002.

When the measurement point was offset in IEC X from center by −2.0 cm, the measured MLC LSPFs were also stable with depth and not jaw‐width dependent. The averaged values obtained were ${\bar{\text{LSPF}}}_{\text{1.0 cm, -2.0 cm}}=0.980\pm 0.004$ and ${\bar{\text{LSPF}}}_{\text{2.5 cm, -2.0 cm}}=0.978\pm 0.003$ for the 1cm and 2.5 cm jaw, respectively. The LSPFs at off‐center locations were also calculated using the measured transverse profiles. An example for 1 cm jaw with −10 mm lateral offset is shown in Fig. 4(c) for two depths (15 and 100 mm) with a few measured points superimposed.

For all Synchrony plans delivered, measured dose outputs, after accounting for the radiograph contribution, are shown in Table I. When the motion amplitude was set to ±20 mm for 1 cm jaw width, the outputs were lower than those of non‐Synchrony plans.

**Table I:** Output measurement results for a series of motion forms for Synchrony vs non‐Synchrony deliveries. The last column is the average of the two ion chamber measurements.

Jaw (cm)	With fiducial	Plan	Motion amplitude (mm)	Output ratio of Synchrony and non‐Synchrony delivery
1.0	Yes	FNR	Irregular up to ±10 mm	1.012
		FR	±20	0.970
			±15	1.007
			±10	1.002
			±5	1.010
	No	FFR	±20	0.957
			±10	1.001
2.5	Yes	FNR	Irregular up to ±10 mm	0.990
				0.990
		FR	±10	1.010
			±5	1.002
	No	FFR	±10	1.001
			±5	0.989

### 3.C. Target detection accuracy in motion monitoring

For the fiducial tracking (without respiratory modeling) with the Sun Nuclear Daily QA3 device, there was no failure in fiducial finding and the delivery was smooth without any interruption. The Potential Diff was stable at ~0.2 mm. The Target Offset was also stable at ~6.8 mm, close to the expected value of 7.1 mm. The Rigid Body was stable at ~0.3 mm. The predicted IEC X, Y, and Z positions were −4.70 ± 0.05, −4.96 ± 0.06, and 0.26 ± 0.06 mm, consistent with the manual offsets within the setup tolerances. The center of motion deviation was −4.9 mm, consistent with the IEC Y offset of −5 mm.

**Fig. 5:**
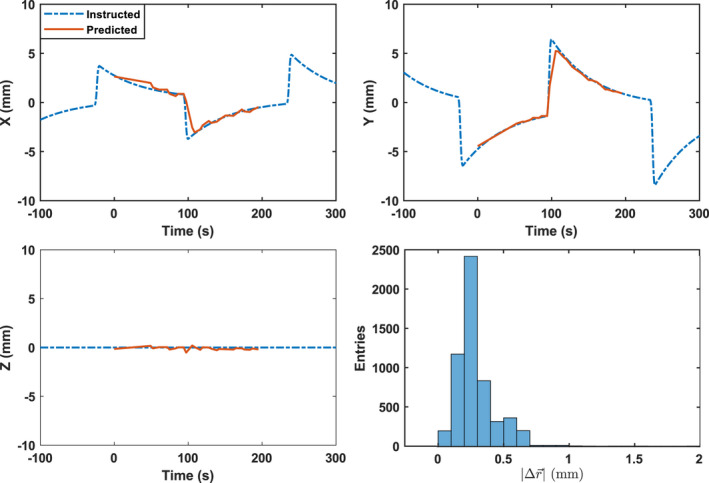
Comparison of instructed and predicted tracking target positions in X, Y, and Z, together with the distribution of three‐dimensional distance between instructed and predicted target positions for the fiducial tracking. [Color figure can be viewed at wileyonlinelibrary.com]

**Fig. 6:**
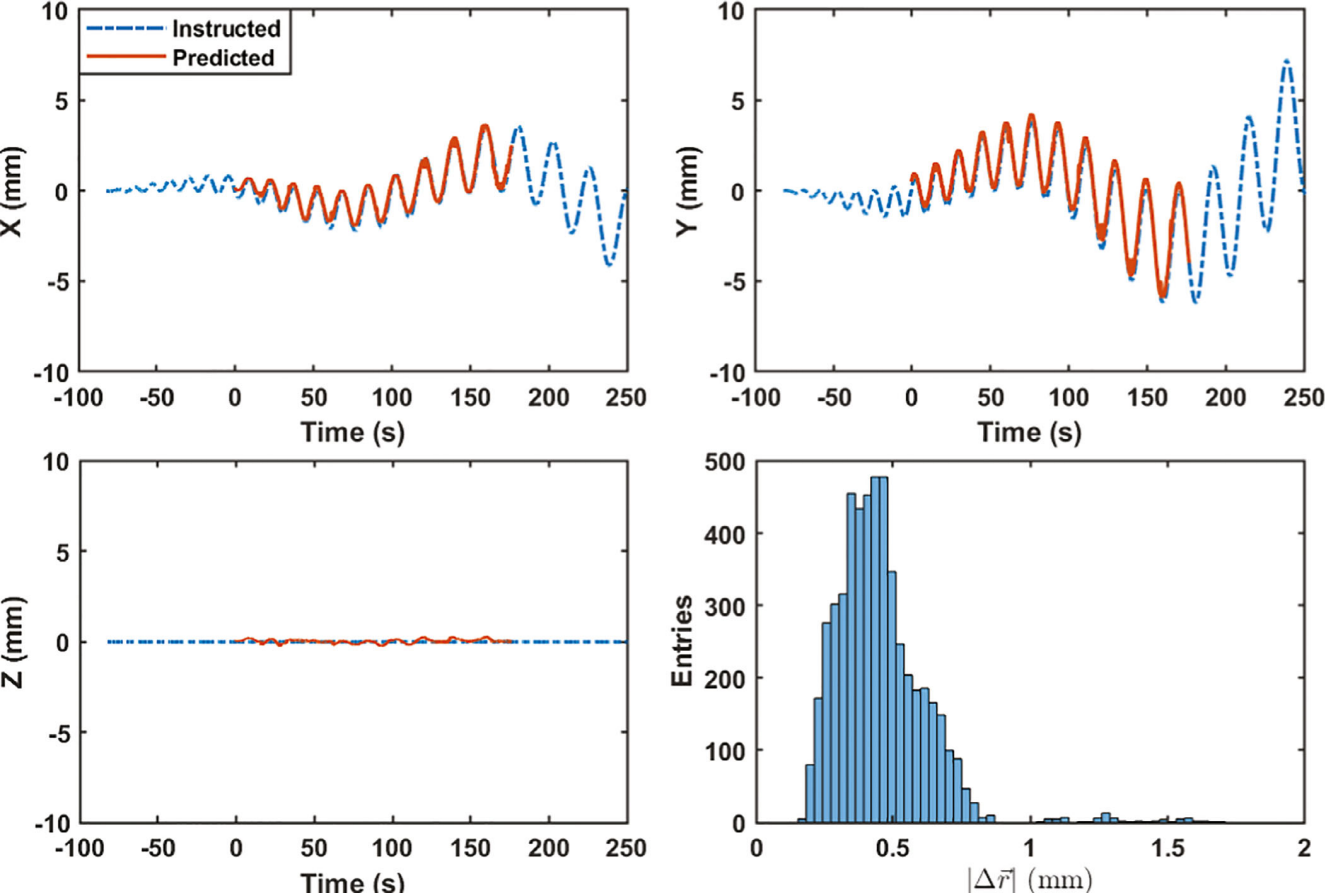
Comparison of instructed and predicted tracking target positions in X, Y, and Z, together with the distribution of three‐dimensional distance between instructed and predicted target positions for the lung tumor tracking. [Color figure can be viewed at wileyonlinelibrary.com]

The comparison of instructed and predicted phantom positions in IEC X, Y, and Z were plotted vs time and the 3D error between the predicted and instructed position are shown in Figs. 5 and 6 for the Synchrony FNR and FFR deliveries, respectively. The result for Synchrony FR (not shown) was similar to that in Fig. 6. For the Synchrony FNR, FR, and FFR deliveries on to the TomoPhantom, the RMS values obtained were 0.84, 1.13, and 0.48 mm, respectively.

### 3.D. Residual latency

**Fig. 7:**
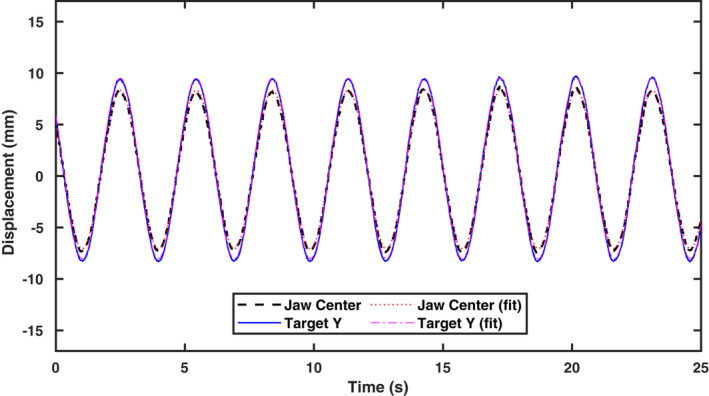
The measured jaw center and predicted target Y positions from the FFR plan delivery. [Color figure can be viewed at wileyonlinelibrary.com]

The variation of the jaw center position, as well as the predicted target Y position with delivery time for the residual latency test obtained with the FFR plan is shown in Fig. 7. The jaw center position and predicted target Y position both followed the sine waves quite well with an RMS of 0.2 mm. The amplitudes, periods, and initial phase time offsets were (7.692 mm, 2.945 s, 1.183 s) and (8.768 mm, 2.945 s, 1.185 s) for the jaw center and target Y positions, respectively. The obtained amplitude for target motion in Y was consistent with the input amplitude of 10 mm with a motion platform rotation of roughly 30°. The amplitude ratio of target to jaw was 1.140, which is very close to the approximate jaw position to field‐edge position conversion factor of 1.161. The periods were both consistent with the preset value of 3 s. The jaw correction has a lag of only 2 ms to the predicted target position in Y.

### 3.E. Dose delivery accuracy with motion compensation

For the five deliveries of the FNR plan on the Daily QA3 device with offset of −5 mm in IEC X and Y, the point doses measured were 99.85, 99.78, 98.30, 99.87, and 97.9 cGy, with an average of 99.1 ± 0.96 cGy, within 2% from the anticipated value of 100.0 cGy.

**Fig. 8:**
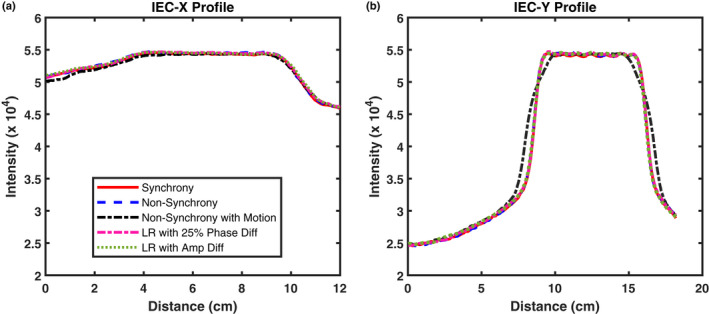
Comparison of IEC X (a) and IEC Y (b) profiles measured with films in the following deliveries: Synchrony with ±10 mm target motion, non‐Synchrony, non‐Synchrony with ±10 mm target motion, Synchrony with ±10 mm target motion but the surrogate with a phase shift of 25%, Synchrony ±10 mm target motion but the surrogate with ±15 mm motion. Big motions were in Y direction. [Color figure can be viewed at wileyonlinelibrary.com]

Figure 8 presents the beam profiles measured with Gafchromic films for five distinct deliveries with respiratory motion corrected in all Synchrony deliveries. The measurements of the non‐Synchrony deliveries with and without motion demonstrate that there was a broadening of the radiation field in the IEC Y direction, the primary direction of the motion, illustrating that without motion correction, the motion‐induced dose difference can be substantial, for example, there are hotspots outside of the target and cold spots within the target. The broadened profile was rectified by implementing Synchrony motion correction. The robustness of Synchrony tracking was further investigated by changing the phase relationship of the target and surrogate motion and the amplitude ratio of the target and surrogate. Surrogate phase shift or amplitude difference between surrogate and target do not affect the motion‐compensated delivery.

**Fig. 9:**
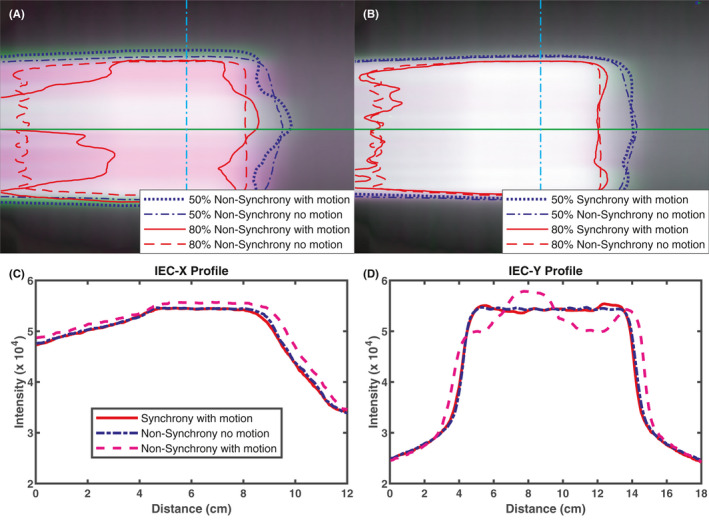
Comparisons of two‐dimensional dose and profile distributions measured with films for the FNR plan deliveries. (a) No motion compensation vs no motion, (b) Motion compensated vs no motion, (c) X profiles, and (d): Y profiles. The profiles were for fiducial tracking with ±10 mm irregular target motion, non‐Synchrony and non‐Synchrony with ±10 mm irregular target motion. Location of profiles is indicated with horizontal solid green (X) and vertical dot dashed cyan (Y) lines on top. [Color figure can be viewed at wileyonlinelibrary.com]

The measurements for fiducial tracking deliveries with nonrespiratory motions are shown in Fig. 9. Comparison of films acquired for the non‐Synchrony plans with and without motion reveal a similar broadening in the IEC Y direction, however, the nature of the motion trace enables realization of the pronounced deviation of the two plans in the IEC X direction [Fig. 9(a)]. When motion compensation is implemented, we see a dramatic improvement in plan agreement [Fig. 9(b)]. A line scan comparison of all three films shows the agreement between the Synchrony and non‐Synchrony plan and the variation when motion is not accounted for in the non‐Synchrony with motion plan [Figs. 9(c) and 9(d)].

The gamma analyses for various 3D dose measurements from the ArcCheck with the criteria of 2 mm, 3% and 10% threshold are shown in Table II. It is seen that the comparisons of the measured to planned dose showed gamma passing rates higher than 95%. The comparison of Synchrony delivery with motion to that without motion also had gamma passing rates of above 95%.

**Table II:** Gamma analyses of ArcCheck measurements with the criteria of 2 mm, 3% and 10% threshold.

Plans	Difference (%) between measured and planned doses			Difference (%)	
	Synchrony with motion	Synchrony no motion	Non‐Synchrony no motion	Synchrony with motion vs Synchrony no motion	Synchrony with motion vs non‐Synchrony no motion
Plan 1	95.7	96.1	99.3	99.3	98.4
Plan 2	99.6	100.0	100.0	100.0	99.7

For FFR deliveries, the gamma passing rates using ArcCheck were 98.3, 95.3, and 100.0% for deliveries of three Synchrony plans based on the CT sets of TomoPhantom, Delta4, and a lung patient, respectively.

With the criteria of 2 mm, 3% and 10% threshold, the gamma passing rates using the Delta4 for plans generated on the Phantom + images were 86.5 (97.2 if 4 mm, 5% with 5% threshold criteria was used), 99.6, and 100.0% for the FNR, FR, and FFR deliveries, respectively. Measurements using Delta4 as a patient plan QA device for Synchrony plans generated on the ArcCheck were obtained, and the gamma passing rates were 99.8, 99.4, and 100.0% for the FNR, FR, and FFR plans, respectively. Note that the subtraction of radiograph dose for diode measurements in gamma analysis was crucial. Without the subtraction, the passing rates were below 85% for FNR and 95% for FR and FFR.

The results with motion jump within jaw range were obtained. For the nonrespiratory motions, the gamma passing rate was 89.9% when the delivery was not paused, which increased to 92.4% when the delivery was resumed after a couch move in Y of the amount determined by the system. For the Synchrony deliveries with respiratory motion and sine wave baseline shift, the passing rates were 99.3% for no pause and 100.0% for a pause with using “Couch Move in Y.”

## 4 DISCUSSION

A comprehensive performance test for the first clinical system of Synchrony on Radixact was carried out to ensure the accurate, effective, and safe use of the system for patients. The system primarily corrects for translational or centroid motion by dynamically repointing each beam projection in a plan sinogram according to the difference between the predicted and planned target positions perpendicular to the beam axis. Neither deformation nor rotation is considered. Our tests confirm that, for commonly encountered motions in clinic, Synchrony on Radixact can adequately compensate by using a variety of adjustable motion tracking parameters.

The additional imaging dose from the motion monitoring using kV radiographs is negligible compared to that from image guidance for radiation delivery. Compared with alternative approaches for real‐time nonionizing radiation motion monitoring, for example, implanted electromagnetic transponders (Calypso),^21^ ultrasound,^35^ and magnetic resonance imaging,^25^ imaging dose using kV x‐ray radiograph is not necessarily a major concern.

As an unflattened beam is used in the Radixact system, the use of off‐axis beams during jaw swing may result in changes in beam characteristics. With motions (<±1 cm) typically observed in most clinical situations, this off‐axis effect is small (<2%). For rare, extremely large motion, for example, 2‐4 cm in the IEC Y direction, the off‐axis effect can be >2%. For oscillatory tumor motion, the target moves to the extreme positions only for a small portion of time during the delivery, resulting in a reduced overall off‐axis effect. It is important to note that large tumor motion, especially with semipermanent tumor translocation to an extreme position, could introduce dose error of a few percent or more. Intervention using the supplied functionality in Synchrony, “Couch Move in Y,” can be used to prevent this error.

Small setup errors can be corrected with Synchrony tracking, likely reducing reliance on setup procedures and/or limiting the need for sizeable setup margins. However, big setup errors should be avoided, especially when the tumor motion range is large, as this would lead to jaw swing out of allowable range as well as noticeable dose error.

Target identification in our tests were quite smooth most of the time. Although not an issue with the performance of Synchrony as designed, we did find that for FNR and FR using the Sun Nuclear ArcCheck, the diodes could be interpreted as fiducials thus could fail to detect the fiducials. As such, integration of Synchrony with common QA tools should be verified independently. For patient treatment, the advantage of fiducial free tracking is that it is noninvasive. However, it may have requirements on size, location of the solid lesions, as well as the density difference relative to the surrounding lung. To verify the robustness of fiducial free tracking, we tested the detection of a simulated aluminum target inserted into the ArcCheck by blocking the target with metal bars from some directions, as well as the diodes inside the ArcCheck. The target detection was successful with carefully selected radiograph image angles to avoid blocking of the target by other big high‐density objects.

The target detection accuracy of Synchrony with and without an internal–external correlation is well within the 1.5 mm suggested tolerance for most of clinical situations. For sudden, large, motions (Fig. 5), the motion detection accuracy can be poor due to the in‐frequent radiographs. For respiratory motion, however, the optical monitoring of the LEDs provides high‐temporal resolution measurements that may improve the motion detection accuracy through the correlation to the internal motion including large, sudden jumps.

The residual latency for respiratory motion compensation, which can be important when considering the model prediction frequency, is small. For irregular motion, the predicted target position is only updated after the acquisition of each radiograph. In such a situation, the target tracking accuracy may be affected. This can be seen from the lower gamma passing rates in Delta4 measurements with FNR plan deliveries. As recommended by the vendor, slightly loose gamma criteria (4 mm 5% with 5% threshold) could be used for fiducial tracking with irregular motion as no predicted target position, until the next radiograph image, was used. The residual latency for irregular motion compensation is expected to be similar to that of respiratory motion compensation, however, it is substantially shorter than the radiograph time, resulting in only minimal contribution to the prediction error. Despite these sources of error, our 2D and 3D measurements for FNR have qualitatively and quantitively demonstrated significant advantages in dose delivery with the motion synchronization.

## 5 CONCLUSIONS

The motion monitoring and compensation with the first clinical Synchrony on Radixact system performed according to the specifications and was effective for motion tracking and compensation. The target detection accuracy was within 1.5 mm for all three available synchronization modes. For all the Synchrony motion compensation deliveries tested, the point dose accuracy was within 2% and the gamma passing rates of 3D doses were within the clinically acceptable range.

## CONFLICT OF INTEREST

Both Guang‐Pei Chen and X. Allen Li have received speaker honoraria from Accuray Inc.

## References

[1] Deshpande S . To study tumor motion and planning target volume margins using four dimensional computed tomography for cancer of the thorax and abdomen regions. J Med Phys. 2011;36:35–39. 21430857 10.4103/0971-6203.75470 PMC3048953

[2] Hanley J , Debois MM , Mah D , et al. Deep inspiration breath‐hold technique for lung tumors: the potential value of target immobilization and reduced lung density in dose escalation. Int J Radiat Oncol Biol Phys. 1999;45:603–611. 10524412 10.1016/s0360-3016(99)00154-6

[3] Wulf J , Hädinger U , Oppitz U , Olshausen B , Flentje M . Stereotactic radiotherapy of extracranial targets: CT‐simulation and accuracy of treatment in the stereotactic body frame. Radiother Oncol. 2000;57:225–236. 11054527 10.1016/s0167-8140(00)00226-7

[4] Li XA , Stepaniak C , Gore E . Technical and dosimetric aspects of respiratory gating using a pressure‐sensor motion monitoring system. Med Phys. 2006;33:145–154. 16485421 10.1118/1.2147743

[5] Kubo HD , Len PM , Minohara S , Mostafavi H . Breathing‐synchronized radiotherapy program at the university of California Davis cancer center. Med Phys. 2000;27:346–353. 10718138 10.1118/1.598837

[6] Keall PJ , Mageras GS , Balter JM , et al. The management of respiratory motion in radiation oncology report of AAPM Task Group 76. Med Phys. 2006;33:3874–3900. 17089851 10.1118/1.2349696

[7] Schweikard A , Shiomi H , Adler J . Respiration tracking in radiosurgery. Med Phys. 2004;31:2738–2741. 15543778 10.1118/1.1774132

[8] Seppenwoolde Y , Berbeco RI , Nishioka S , et al. Accuracy of tumor motion compensation algorithm from a robotic respiratory tracking system: a simulation study. Med Phys. 2007;34:2774–2784. 17821984 10.1118/1.2739811

[9] D'Souza WD , Naqvi SA , Yu CX . Real‐time intra‐fraction‐motion tracking using the treatment couch: a feasibility study. Phys Med Biol. 2005;50:4021–4033. 16177527 10.1088/0031-9155/50/17/007

[10] Qiu P , D’Souza WD , McAvoy TJ , et al. Inferential modelling and predictive feedback control in real‐time motion compensation using the treatment couch during radiotherapy. Phys Med Biol. 2007;52:5831–5854. 17881803 10.1088/0031-9155/52/19/007

[11] Adler JR Jr , Chang SD , Murphy MJ , Doty J , Geis P , Hancock SL . The cyberknife: a frameless robotic system for radiosurgery. Stereotact Funct Neurosurg. 1997;69:124–128. 9711744 10.1159/000099863

[12] Ozhasoglu C , Saw CB , Chen H , et al. Synchrony – Cyberknife respiratory compensation technology. Med Dosim. 2008;33:117–123. 18456163 10.1016/j.meddos.2008.02.004

[13] Kamino Y , Takayama K , Kokubo M , et al. Development of a four‐dimensional image‐guided radiotherapy system with a gimbaled x‐ray head. Int J Radiat Oncol Biol Phys. 2006;66:271–278. 16820270 10.1016/j.ijrobp.2006.04.044

[14] Takayama K , Mizowaki T , Kokubo M , et al. Initial validations for pursuing irradiation using a gimbals tracking system. Radiother Oncol. 2009;93:45–49. 19699544 10.1016/j.radonc.2009.07.011

[15] Ziegler M , Brandt T , Lettmaier S , Fietkau R , Bert C . Performance of gimbal‐based dynamic tumor tracking for treating liver carcinoma. Radiat Oncol. 2018;13:1–12. 30518398 10.1186/s13014-018-1180-1 PMC6280466

[16] Keall PJ , Sawant A , Cho B , et al. Electromagnetic‐guided dynamic multileaf collimator tracking enables motion management for intensity‐modulated arc therapy. Int J Radiat Oncol Biol Phys. 2011;79:312–320. 20615630 10.1016/j.ijrobp.2010.03.011 PMC2953612

[17] Keall PJ , Kini VR , Vedam SS , Mohan R . Motion adaptive x‐ray therapy: a feasibility study. Phys Med Biol. 2001;46:1–10. 11197664 10.1088/0031-9155/46/1/301

[18] Keall PJ , Cattell H , Pokhrel D , et al. Geometric accuracy of a real‐time target tracking system with dynamic multileaf collimator tracking system. Int J Radiat Oncol Biol Phys. 2006;65:1579–1584. 16863935 10.1016/j.ijrobp.2006.04.038

[19] Sawant A , Venkat R , Srivastava V , et al. Management of three‐dimensional intrafraction motion through real‐time DMLC tracking. Med Phys. 2008;35:2050–2061. 18561681 10.1118/1.2905355 PMC2809733

[20] Sawant A , Smith RL , Venkat RB , et al. Toward submillimeter accuracy in the management of intrafraction motion: the integration of real‐time internal position monitoring and multileaf collimator target tracking. Int J Radiat Oncol Biol Phys. 2009;74:575–582. 19327907 10.1016/j.ijrobp.2008.12.057

[21] Keall PJ , Colvill E , O’Brien R , et al. The first clinical implementation of electromagnetic transponder‐guided MLC tracking. Med Phys. 2014;41:020702. 24506591 10.1118/1.4862509 PMC3977852

[22] Tacke MB , Nill S , Krauss A , Oelfke U . Real‐time tumor tracking: automatic compensation of target motion using the Siemens 160 MLC. Med Phys. 2010;37:753–761. 20229885 10.1118/1.3284543

[23] Krauss A , Nill S , Tacke M . Oelfke U . Electromagnetic real‐time tumor position monitoring and dynamic multileaf collimator tracking using a Siemens 160 MLC: geometric and dosimetric accuracy of an integrated system. Int J Radiat Oncol Biol Phys. 2011;79:579–587. 20656420 10.1016/j.ijrobp.2010.03.043

[24] Fast MF , Nill S , Bedford JL , Oelfke U . Dynamic tumor tracking using the Elekta agility MLC. Med Phys. 2014;41:111719. 25370633 10.1118/1.4899175

[25] Crijns SPM , Raaymakers BW , Lagendijk JJW . Proof of concept of MRI‐guided tracked radiation delivery: tracking one‐dimensional motion. Phys Med Biol. 2012;57:7863–7872. 23151821 10.1088/0031-9155/57/23/7863

[26] PRNewswire.com . Chicago, IL: Cision Ltd https://www.prnewswire.com/news‐releases/accuray‐launches‐synchrony‐motion‐tracking‐and‐correction‐technology‐for‐the‐radixact‐system‐300836858.html

[27] Kilby W , Dooley J , Kuduvalli G , Sayeh S , Maurer C Jr . The CyberKnife robotic radiosurgery system in 2010. Technol Cancer Res Treat. 2010;9:433–452. 20815415 10.1177/153303461000900502

[28] Schnarr E , Beneke M , Casey D , et al. Feasibility of real‐time motion management with helical tomotherapy. Med Phys. 2018;45:1329–1337. 29405307 10.1002/mp.12791

[29] Chao E , Lucas D , Schnarr E . Evaluation of TomoTherapy dose calculations with intrafractional motion and motion compensation. Med Phys. 2018;45:18–28. 29106739 10.1002/mp.12655

[30] Accuray.com . Sunnyvale, CA: Accuray Inc http://investors.accuray.com/news‐releases/news‐release‐details/froedtert‐medical‐college‐wisconsin‐cancer‐network‐first‐world

[31] Bauhs JA , Vrieze TJ , Primak AN , Bruesewitz MR , McCollough CH . CT Dosimetry: comparison of measurement techniques and devices. Radiographics. 2008;28:245–253. 18203941 10.1148/rg.281075024

[32] Yarahmadi M , Wegener S , Sauer OA . Energy and field size dependence of a silicon diode designed for small‐field dosimetry. Med Phys. 2017;44:1958–1964. 28273357 10.1002/mp.12195

[33] Miften M , Olch A , Mihailidis D , et al. Tolerance limits and methodologies for IMRT measurement‐based verification QA: recommendations of AAPM Task Group No. 218. Med Phys. 2018;45:e53–e83. 29443390 10.1002/mp.12810

[34] Accuray training document 1064193.A . Synchrony® Real‐Time Motion Tracking and Correction on the Radixact® Treatment Delivery System. Sunnyvale, CA: Accuray Inc, 2019.

[35] Schlosser J , Salisbury K , Hristov D . Telerobotic system concept for real‐time soft‐tissue imaging during radiotherapy beam delivery. Med Phys. 2010;37:6357–6367. 21302793 10.1118/1.3515457

